# Exploration of metabolite profiles in the biofluids of dairy cows by proton nuclear magnetic resonance analysis

**DOI:** 10.1371/journal.pone.0246290

**Published:** 2021-01-29

**Authors:** Hyun Sang Kim, Eun Tae Kim, Jun Sik Eom, You Young Choi, Shin Ja Lee, Sang Suk Lee, Chang Dae Chung, Sung Sill Lee

**Affiliations:** 1 Division of Applied Life Science (BK21Four), Gyeongsang National University, Jinju, Korea; 2 National Institute of Animal Science, Rural Development Administration, Cheonan, Korea; 3 Institute of Agriculture and Life Science & University-Centered Labs, Gyeongsang National University, Jinju, Korea; 4 Ruminant Nutrition and Anaerobe Laboratory, College of Bio-industry Science, Sunchon National University, Suncheon, Korea; University of Illinois, UNITED STATES

## Abstract

Studies that screen for metabolites produced in ruminants are actively underway. We aimed to evaluate the metabolic profiles of five biofluids (ruminal fluid, serum, milk, urine, and feces) in dairy cow by using proton nuclear magnetic resonance (^1^H-NMR) and provide a list of metabolites in each biofluid for the benefit of future research. We analyzed the metabolites in five biofluids from lactating cows using proton nuclear magnetic resonance imaging; 96, 73, 88, 118, and 128 metabolites were identified in the five biofluids, respectively. In addition, 8, 6, 9, and 17 metabolites were unique to ruminal fluid, serum, milk, and urine, respectively. The metabolites present at high concentrations were: acetate, propionate, and butyrate in ruminal fluid; lactate, glucose, and acetate in serum; and lactose, guanidoacetate, and glucitol in milk. In addition, the following metabolites were present at high concentrations: hippurate, urea, and trimethylamine N-oxide in urine and acetate, propionate, and butyrate in feces. The score plots of the principal component analysis did not show clear distinctions among the five biofluid samples. The purpose of this study was to verify the ability of our metabolomics approaches to identify metabolites in the biofluids of dairy cows.

## Introduction

Metabolomics studies directly reflect the metabolic pathways involved in the production of metabolites and yield essential information regarding the underlying biological system [1]. They mainly involve processing of biofluids such as blood, urine, and fecal samples, followed by analysis of metabolites from various metabolic pathway matrices and products [2]. There are many examples of metabolomics research on human biofluids and tissues, as well as, on animals including experimental animals such as mice and livestock [3].

A variety of methods, including high pressure liquid chromatography and gas chromatography–mass spectrometry (GC-MS), and nuclear magnetic resonance (NMR) can be used as representative instruments for metabolic analyses [4]. The major advantages of MS are its high sensitivity and that it can be used to measure hundreds of metabolites. However, its disadvantages include that the quantity of the sample has to be high and it lacks reproducibility and reliability. On the other hand, NMR is easy to appropriate and has high reproducibility and reliability, but the sensitivity is relatively low. Therefore, NMR-based metabolic studies are suitable for the quick identification of metabolic differences between groups and the study of metabolic pathways that involve relatively high concentrations of metabolites. In addition, NMR has the advantage of easily identifying metabolites from analysis data using databases and libraries [5].

Metabolomics studies on livestock have increased rapidly, especially in the last few years. Across all livestock categories, the most extensive metabolomics studies have been conducted on cattle [6]. Commonly used biofluids include ruminal fluid, serum, milk, and urine. These studies are also conducted to assess disease risk biomarkers and metabolic diseases such as ketosis and rumen acidosis [7–10], feeding roughage type [11], improvement of milk productivity [12], and generation of a ruminal fluid and serum metabolome database [13, 14]. Studies using various biofluids have examined lactation-related metabolic mechanisms and potential biomarkers for milk production and quality [12, 15]. However, the analysis of metabolites in different biofluids of a single animal has only been conducted in a few studies [15–17]. Furthermore, little is known about the general concentration range of important metabolites in the biofluids of dairy cows.

Therefore, the current study aimed to use proton NMR (^1^H-NMR) metabolomics to investigate the ruminal fluid, serum, milk, urine, and feces from dairy cows for non-targeted metabolite profiling. This study provides potentially relevant information related to the types of common metabolites present in the biofluids of dairy cows. Moreover, the results of this study provide a useful database for the analysis of metabolites in ruminant biofluids.

## Materials and methods

This study was carried out in strict accordance with the recommendations in the Guide for the Care and Use of Laboratory Animals of the National Institute of Animal Science in Korea. The protocol was approved by the Committee on the Ethics of Animal Experiments of the National Institute of Animal Science (NIAS-201908).

### Experimental animals and sample collection

Four Holstein cows (body weight: 565 ± 41 kg; days since calving: 170 ± 16; parity: 1) from the National Institute of Animal Science were used in this study. Dairy cows were fed 8 kg of Italian ryegrass hay and 2 kg of concentrates twice a day on a dry matter basis and given *ad libitum* access to mineral blocks and water. The chemical composition of the feed is shown in the S1 Table.

Ruminal fluid was taken from the four-cows using a stomach tube. The first ruminal fluid was not sampled due to saliva and blood contamination. The pH of ruminal fluid was measured immediately after collection using a pH meter (MP230, Mettler-Toledo, Columbus, OH, USA). Thereafter, the ruminal fluid samples were centrifuged at 810 × *g* for 15 min to remove feed particles and the supernatants were stored at -80°C until further metabolite analysis. Urine samples were collected by stimulating the cow to urinate by sweeping the perineum by hand and stored at -80°C. Feces were sampled from the hind-gut by hand. Blood from the neck jugular vein of each cow was collected in serum tubes. Serum samples were centrifuged for 5 min at 15,140 × *g* and aliquots of the upper layer (plasma) were immediately frozen. Samples were stored at -80°C. Raw milk samples were collected from individual animals using a milking machine.

### Sample preparation for NMR spectroscopy

The ruminal samples were centrifuged at 12,900 × *g* for 10 min and the supernatant was collected (300 μL). Standard buffer solution (TSP; 2,2,3,3-d(4)-3-(Trimethylsilyl)propionic acid sodium salt) was added to 300 μL of supernatant in deuterium oxide (D_2_O) solvent/standard buffer solution (300 μL). This mixture was transferred to a 5 mm NMR tube for NMR spectral analysis. This sample pre-treatment method was performed with reference to Saleem et al. [13].

A saline buffer with NaCl 0.9% weight/volume in 100% D_2_O was prepared. The stored serum sample was centrifuged at 14,000 × *g* for 10 min at 4°C. The supernatant (200 μL) was added to 400 μL of saline buffer in the 5 mm NMR tube for measurement. An ERETIC peak reference sample was also prepared by adding 388 μL of saline solution and 12 μL of 100 mM valine solution to 200 μL of the supernatant to yield a final valine concentration of 2 mM, which was then transferred to the 5 mm NMR tube. This sample pre-treatment method was performed with reference to Jung et al. [18].

The collected milk sample was centrifuged at 4,000 × *g* for 15 min to remove the lipid layer in the supernatant. Thereafter, the mixture of milk (250 μL) and D_2_O (300 μL) was transferred to a 5 mm NMR tube for analysis. This sample pre-treatment method was performed with reference to Lamanna et al. [19].

The urine sample was added to 0.2 M sodium phosphate buffer (pH 7.0) and centrifuged to collect 400 μL of supernatant at 14,000 × *g* for 10 min at 4°C. The supernatant was added to 230 μL of buffer and was adjusted to pH 7.0 ± 0.1. The mixture was added to 2 mM TSP (60 μL) and TSP concentration in the total solution was adjusted to 0.2 mM. The prepared sample was transferred to a 5 mm NMR tube for NMR spectral analysis. This sample pre-treatment method was performed with reference to Jeong et al. [20].

The fecal sample was dried at 50°C in an oven for 1 day. The dried sample (300 mg) was mixed with 1 mL of phosphate-buffered solution (PBS, pH 7.4). PBS was added to sodium chloride (25 mg NaCl/10 mL buffer solution) to facilitate extraction. The mixture was homogenized using an ultrasonic homogenizer for 15 min and centrifuged at 14,230 × *g* for 20 min. The supernatant (400 μL) was mixed with 150 μL of D_2_O and added to 50 μL of 5 mM TSP. After secondary centrifugation, the supernatant of the mixture was transferred to an 5 mm NMR tube and used for NMR analysis. This sample pre-treatment method was performed with reference to Deda et al. [21].

### Proton NMR analyses

The spectra were measured using AVANCE III HD Bruker spectrometer (Bruker BioSpin AG, Fällanden, Switzerland). ^1^H-NMR spectra were acquired at 25°C using the first transient nuclear overhauser effect spectroscopy pre-saturation pulse sequence (ruminal fluid, milk, urine, and fecal samples) and the Carr-Purcell-Meiboom-Gill pulse sequence (serum sample). Spectra were collected with 128 transients using an acquisition time of 2 s. A spectral width of 20 ppm was used to collect 64,000 data points. Processed spectra were imported into the Chenomx NMR suite 8.4 software (Chenomx Inc, Edmonton, Canada). All ^1^H NMR spectra were adjusted step-by-step under manual calibration using a module of Chenomx NMR software, Chenomx Processor, corrected baseline, and calibrated (DSS or TSP 0.0 ppm). Peaks in the ^1^H NMR spectrum were assigned, with reference, to known chemical shifts and peak multiplicity in relation to the Chenomx 800-MHz library database. Metabolite quantification was performed using Chenomx Profiler, another module included in the Chenomx NMR Suite software, which evaluates the levels of individual metabolites using the concentrations of know chemical shape indicators (DSS and TSP) signals. The spectral width was 10 ppm and was referenced to the TSP and DSS signal at 0 ppm. The databases of metabolites used were the Livestock Metabolite Database (www.lmdb.ca), Bovine Metabolite Database (www.bmdb.ca), and Chenomx library. The classification and quantity of metabolites were used in the Chenomx profiler program.

### Statistical analyses

Statistical analyses of metabolite data were conducted using MetaboAnalyst 4.0, an open-source R-based program for metabolomics. Data were normalized by sum, log transformation, and pareto scaling before statistical analyses. Principal component analysis (PCA) and partial least squares-discriminant analysis (PLS-DA) were used for multivariate data analyses to generate a classification model and provide quantitative information on metabolites. Further validation was performed with MetaboAnalyst 4.0, using permutation tests with 1000 permutations. The important classifiers were identified via their variable importance in projection (VIP) scores. Metabolic pathway analysis was performed using a list of the top 30 metabolites and the results with p-values of less than 0.05 are shown in the figures.

## Results

**Fig 1:**
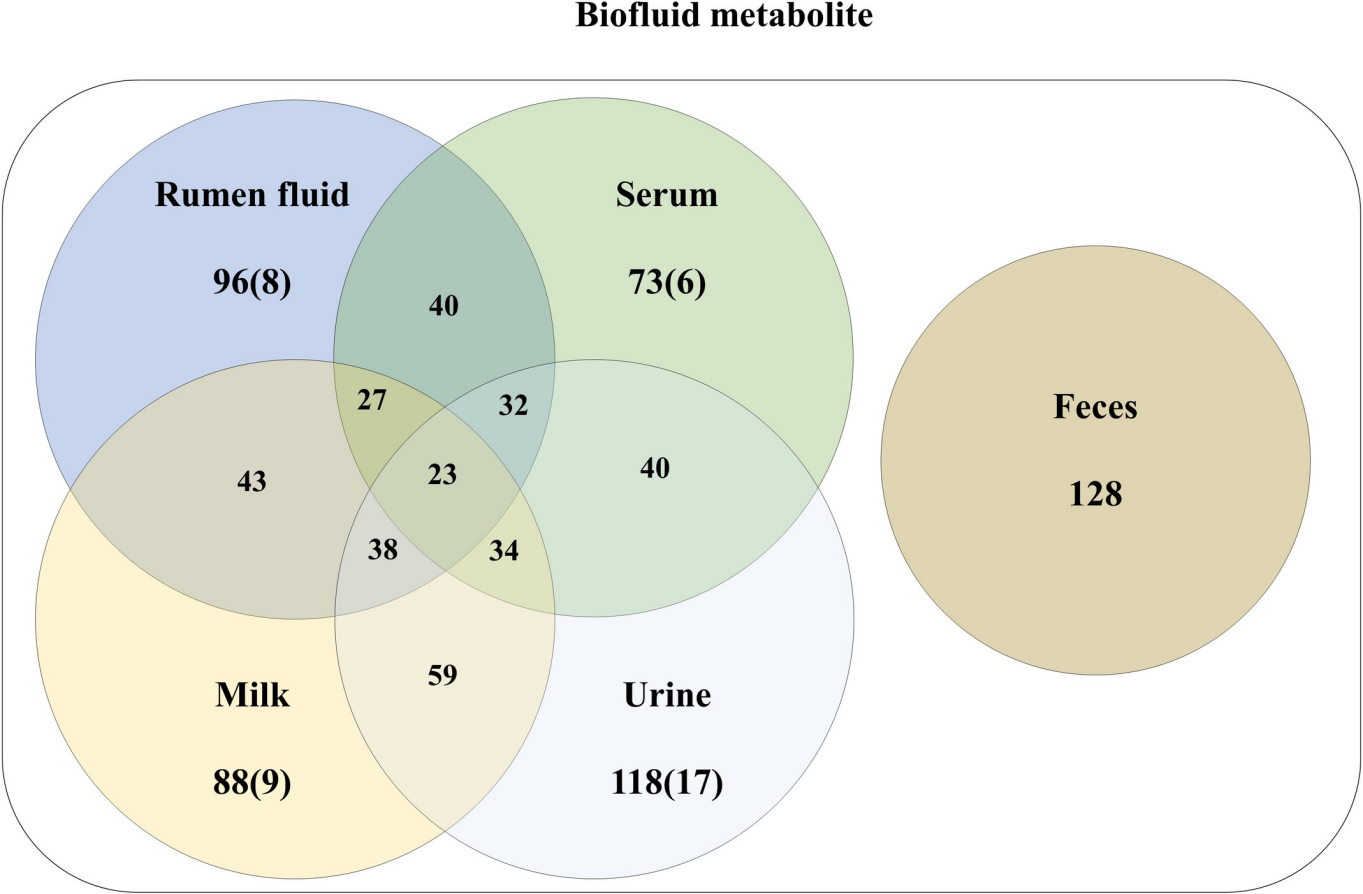
The overlap of biofluids metabolites by ^1^H-NMR compared to the detectable metabolome. The overlap of metabolites is represented by a Venn diagram of metabolites identified in biofluids in at least three samples within each group. The number in the circle indicates the metabolites detected in the respective biofluids and the number in parentheses represents the unique metabolites in each biofluid.

**Fig 2:**
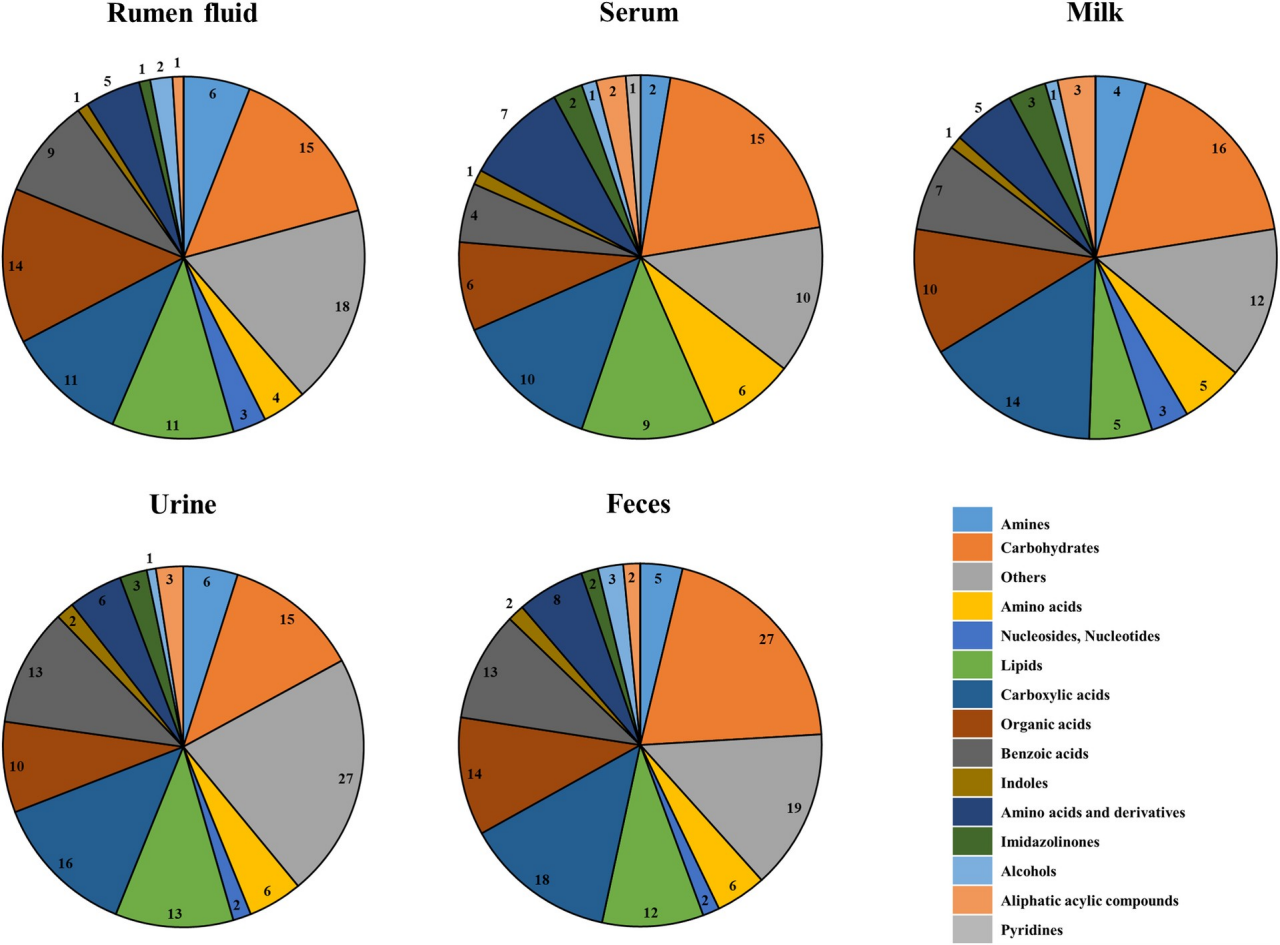
The classification of identified metabolites according to chemical class in biofluids.

**Fig 3:**
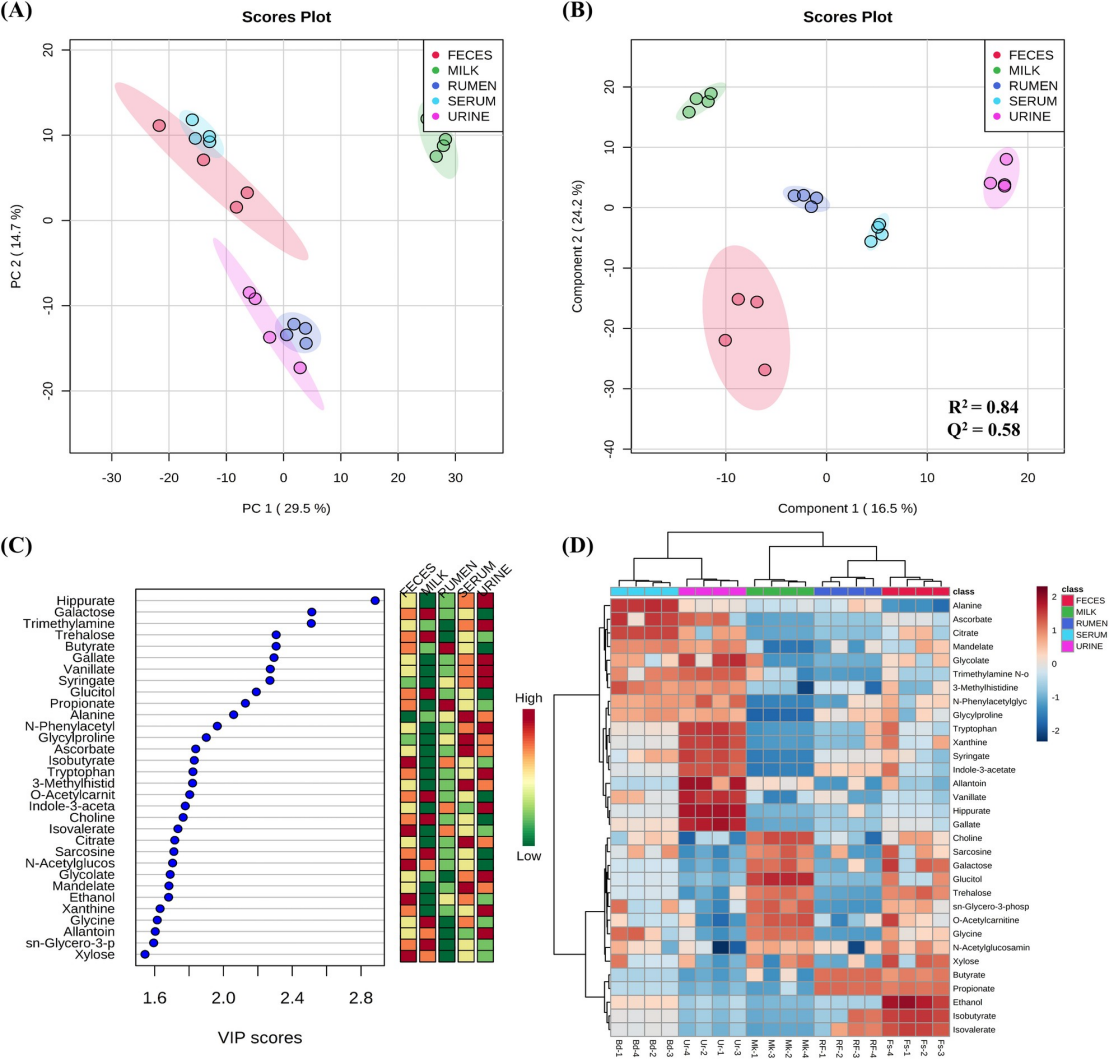
Multivariate data analysis of non-targeted metabolites of biofluids. (A) PCA score, (B) PLS-DA score plot, (C) PLS-DA VIP Scores of top 32 metabolites with Scores > 1.5, (D) Heat map of the metabolite profiles based on the PLS-DA VIP scores.

We investigated the metabolite changes in five biofluids (ruminal fluid, serum, milk, urine, and feces) in the dairy cow by ^1^H-NMR analysis. The metabolic variation across the biological matrices is displayed in a Venn diagram in Fig 1 and S2 Table. The assigned metabolites are indicated in S1 Fig, which shows a typical NMR spectrum of the biofluid. The intensity peaks in the 0.5–9.0 ppm range in the ^1^H-NMR spectra were associated with the presence of various fermentation products, such as organic acids, amino acids, alcohols, lipids, and other organic compounds. The ^1^H-NMR peaks were analyzed using Chenomx NMR suite software for the identification and quantification of metabolites. In addition to the classification of biofluid metabolite profiling, amines, carbohydrates, amino acids, three nucleosides and nucleotides, lipids, carboxylic acids, organic acids, benzoic acids, indoles, amino acids and derivatives, imidazolinones, alcohols, aliphatic acyclic compounds, pyridines, and others were quantified (Fig 2). Multivariate analyses, such as PCA and PLS-DA were used to interpret the results obtained using ^1^H-NMR. The score plots of PCA and PLS-DA are shown in Fig 3A and 3B, respectively. The score plots of PCA did not show a clear separation among the biofluid samples (Fig 3A), with the exception of the milk sample. However, PLS-DA plots clearly separated clusters among all the biofluid samples (Fig 3B). Further model validation relied on a permutation analysis. This analysis was performed using only the best models. The results were considered significant if the performance scores of the original data were outside the distribution. Using 1,000 permutation tests, the p-value was determined as p = 0.001, indicating that the result is significantly better than the original value (S2 Fig). Based on the relative intensities of the metabolites from the normalized spectrum, VIP-score plots constructed from the supervised PLS-DA analysis of biofluid was used to reveal the significant differences of identified metabolites in biofluid (Fig 3C). The results of the heat map based on the PLS-DA result using MetaboAnalyst exhibited different distribution patterns for the metabolites (Fig 3D).

### Ruminal sample

**Fig 4:**
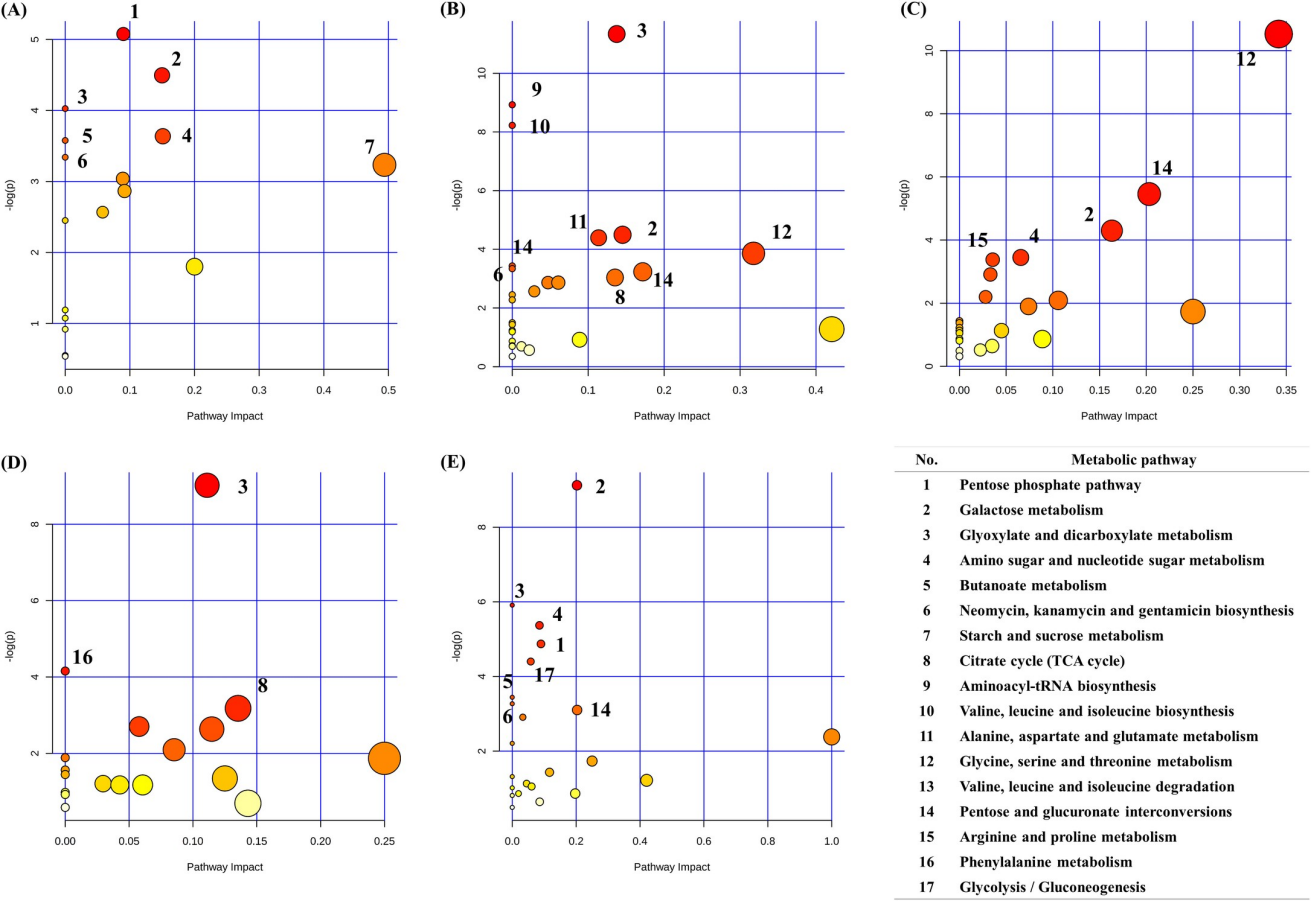
Overview of metabolic pathway analysis. The ‘metabolome view’ presents pathways arranged according to the scores based on enrichment analysis (y-axis) and topology analysis (x-axis). The color and size of each circle is based on p-values and pathway impact values, respectively. Only pathways with p-value lower 0.05 were labelled. (A) Ruminal fluid, (B) Serum, (C) Milk, (D) Urine, (E) Feces.

A total of 96 metabolites was identified in the ruminal sample; just 8 metabolites (Uracil, threonate, pimelate, N-phenylacetylphenylalanine, N-acetylomithine, caprate, etc) of them were identified in ruminal sample (Fig 1 and S2 Table). The average concentration of the top 30 metabolites in the ruminal fluid is shown in Table 1. The ruminal fluid was observed to contain a higher concentration of volatile fatty acids (VFAs) (acetate, propionate, and butyrate) than the other biofluids within the data matrix. The metabolites in the ruminal samples were involved in the pentose phosphate pathway; galactose metabolism; glyoxylate and dicarboxylate metabolism; amino sugar and nucleotide sugar metabolism; butanoate metabolism; neomycin, kanamycin, and gentamicin biosynthesis; and starch and sucrose metabolism (Fig 4A).

**Table 1:** Concentrations of the top 30 metabolites in ruminal fluid by ^1^H-NMR analysis.

Metabolite	Concentration^1^	Metabolite	Concentration
*Alcohols*		*Lipids*	
Methanol	10.37 ± 0.87	Pimelate	46.6 ± 13.00
*Amines*		Caprate	38.67 ± 4.49
Methylamine	142.17 ± 12.69	3-Hydroxybutyrate	12.33 ± 5.80
Dimethylamine	20.73 ± 6.99	2-Hydroxy-3-methylvalerate	11.85 ± 4.15
*Amino acids and derivatives*		3-Hydroxy-3-methylglutarate	10.9 ± 2.98
Alanine	13.62 ± 6.34	*Organic acids*	
*Carbohydrates*		Acetate	13664.2 ± 864.4
Glucose	63.37 ± 25.51	Propionate	2959.7 ± 112.8
Fructose	41.25 ± 7.95	Butyrate	1659.7 ± 190.4
Lactose	38.75 ± 0.55	Valerate	135.83 ± 18.65
Maltose	23.62 ± 5.77	Isobutyrate	82.1 ± 13.69
Glucose-6-phosphate	22.35 ± 6.37	Isovalerate	39.5 ± 12.76
Isocitrate	21.36 ± 3.06	Malate	22.32 ± 3.20
Ribose	19.25 ± 0.65	Gluconate	21.4 ± 5.70
N-Acetylglucosamine	15.4 ± 3.85	*Others*	
Lactulose	12.55 ± 2.39	3-Phenylpropionate	191.9 ± 26.1
*Carboxylic acids*		Biotin	19.72 ± 8.08
Glycylproline	20.95 ± 7.58		
N-Acetylglycine	12.6 ± 1.33		

^1^ μM, Median ± interquartile range (n = 4).

### Serum sample

A total of 73 metabolites was identified in the serum samples; just 6 metabolites (5,6-dihydrothymine, alloisoleucine, glutamine, lactate, ornithine and sucrose) of them were identified in serum sample (Fig 1 and S2 Table). The average concentration of the top 30 metabolites in the serum is shown in Table 2. The concentration of glucose and lactate was higher in the serum than in the other biofluids and the concentration of acetate was lower in the serum than in the ruminal fluid. The metabolites in the serum samples were involved in glyoxylate and dicarboxylate metabolism; aminoacyl-tRNA biosynthesis; valine, leucine, and isoleucine biosynthesis; galactose metabolism; alanine, aspartate, and glutamate metabolism; glycine, serine, and threonine metabolism; valine, leucine, and isoleucine degradation; neomycin, kanamycin, and gentamicin biosynthesis; pentose and glucuronate interconversion; and tricarboxylic acid cycle (Fig 4B).

**Table 2:** Concentrations of the top 30 metabolites in serum by ^1^H-NMR analysis.

Metabolite	Concentration^1^	Metabolite	Concentration
*Alcohols*		*Carboxylic acids*	
Methanol	96.17 ± 2.97	Glycylproline	8.72 ± 1.19
*Aliphatic acylic compounds*		Alloisoleucine	5.45 ± 3.65
Trimethylamine N-oxide	45.05 ± 15.98	*Imidazolinones*	
*Amino acids*		Creatinine	12.42 ± 0.49
Creatine	49.25 ± 2.75	*Lipids*	
Glutamine	48.3 ± 2.5	3-Hydroxybutyrate	55.76 ± 9.31
*Amino acids and derivatives*		2-Hydroxyvalerate	23.25 ± 3.75
Alanine	47.55 ± 2.04	3-Hydroxy-3-methylglutarate	7.7 ± 1.22
Glycine	46.26 ± 24.29	Carnitine	6.43 ± 2.4
Valine	34.02 ± 6.4	*Organic acids*	
Isoleucine	16.9 ± 3.8	Lactate	315.7 ± 17.5
Leucine	16.85 ± 3.86	Acetate	153.5 ± 13.1
*Carbohydrates*		Gluconate	13.7 ± 1.5
Glucose	286.6 ± 20.6	Formate	10.65 ± 0.31
Citrate	50.85 ± 1.39	*Others*	
Xylitol	19.4 ± 3.19	Betaine	32.47 ± 1.92
Ribose	17.66 ± 8.4	Arabinose	16.05 ± 6.74
Isocitrate	15.05 ± 2.37	Acetone	6.72 ± 0.21
Mannose	14.67 ± 3.3		
Lactose	6.73 ± 1.44		

^1^ μM, Median ± interquartile range (n = 4).

### Milk sample

A total of 88 metabolites was identified in the milk sample; just 9 metabolites (2-furoylglycine, 2-oxocaproate, arabinitol, glucarate, guanidinosuccinate, inosine, etc.) of them were identified in the milk sample (Fig 1 and S2 Table). The average concentration of the top 30 metabolites in milk is shown in Table 3. Lactose had the highest concentration, followed by guanidoacetate, glucitol, and glycine. The metabolites in the milk sample were involved in glycine, serine, and threonine metabolism; pentose and glucuronate interconversion; galactose metabolism; amino sugar and nucleotide sugar metabolism; and arginine and proline metabolism (Fig 4C).

**Table 3:** Concentrations of the top 30 metabolites in milk by ^1^H-NMR analysis.

Metabolite	Concentration^1^	Metabolite	Concentration
*Aliphatic acylic compounds*		*Carboxylic acids*	
Urea	201.37 ± 3.52	Guanidoacetate	4880.3 ± 138.8
Trimethylamine N-oxide	123.8 ± 120.2	Creatine phosphate	199.47 ± 102.93
*Amino acids*		N-Carbamoylaspartate	97.05 ± 34.35
Creatine	83.6 ± 39.49	5-Aminolevulinate	56.4 ± 22.83
*Amino acids and derivatives*		*Imidazolinones*	
Glycine	1787.6 ± 75.0	Creatinine	110.15 ± 28.1
*Benzoic acids*		*Lipids*	
Tartrate	72.86 ± 3.11	Choline	372.62 ± 39.23
*Carbohydrates*		O-Acetylcarnitine	92.75 ± 9.16
Lactose	43324.3 ± 2799.2	Carnitine	84.82 ± 12.07
Glucitol	3522.7 ± 437.6	*Organic acids*	
Arabinitol	880.85 ± 127.24	Butyrate	59.1 ± 22.7
Glucuronate	704.4 ± 168.3	*Others*	
Isocitrate	297.3 ± 40.4	sn-Glycero-3-phosphocholine	352.3 ± 67.48
Galactose	276.6 ± 76.28	Cellobiose	248.07 ± 8.62
Xylose	137.06 ± 31.58	Betaine	190.42 ± 18.16
N-Acetylglucosamine	126.95 ± 19.33	2-Oxocaproate	120.33 ± 15.12
Trehalose	108.35 ± 10.96	Fucose	73.45 ± 0.75
Lactulose	103.55 ± 34.5		
Ribose	51.2 ± 5.1		

^1^ μM, Median ± interquartile range (n = 4).

### Urine sample

A total of 118 metabolites was identified in the urine sample; just 17 metabolites (Acetoin, cholate, gallate, hippurate, histidine, kynurenate, etc.) of them were identified in urine sample (Fig 1 and S2 Table). The average concentration of the top 30 metabolites in urine is shown in Table 4. Hippurate had the highest concentration, followed by urea, trimethylamine N-oxide, glycolate, and allantoin. The metabolites in the urine sample were involved in glyoxylate and dicarboxylate metabolism; phenylalanine metabolism; and tricarboxylic acid cycle (Fig 4D).

**Table 4:** Concentrations of the top 30 metabolites in urine by ^1^H-NMR analysis.

Metabolite	Concentration^1^	Metabolite	Concentration
*Aliphatic acylic compounds*		*Indoles*	
Urea	4305 ± 1314.1	3-Indoxylsulfate	42.66 ± 18.89
Trimethylamine N-oxide	3053 ± 737.5	*Carbohydrates*	
*Amines*		Lactose	429.2 ± 148.96
Dimethylamine	172.2 ± 98.9	Isocitrate	116.3 ± 64.29
*Amino acids*		Fructose	94.45 ± 52.85
N-Phenylacetylglycine	142.65 ± 42.1	Glucose-6-phosphate	70.5 ± 58.3
1-Methylhistidine	53.42 ± 44.98	Glucuronate	56.95 ± 38.05
*Amino acids and derivatives*		Citrate	51.23 ± 18.63
Hippurate	8384.4 ± 2619.6	*Lipids*	
Tryptophan	52.62 ± 16.4	Glycolate	2776.6 ± 1029.8
*Benzoic acids*		Sebacate	151.1 ± 65.5
Syringate	137.75 ± 48.9	3-Methylglutarate	92.15 ± 24.85
Gallate	133.9 ± 54.63	2-Hydroxyvalerate	72.5 ± 26.79
Vanillate	98.52 ± 33.77	3,5-Dibromotyrosine	66.96 ± 28.98
4-Hydroxyphenylacetate	74.1 ± 14.81	*Nucleosides*, *Nucleotides*	
Gentisate	58.1 ± 54.2	Xanthine	142.2 ± 49.86
*Carboxylic acids*		*Organic acids*	
Glycylproline	112.32 ± 48.68	Acetate	249.53 ± 80.82
*Imidazolinones*		Formate	83.66 ± 26.79
Allantoin	1056.5 ± 495.5		
Creatinine	375.1 ± 201.98		

^1^ μM, Median ± interquartile range (n = 4).

### Fecal sample

A total of 128 metabolites was identified in the fecal sample; just 19 metabolites (Azelate, butanone, ethylene glycol, galactitol, glutamate, glutathione, etc.) of them were identified in the fecal sample (Fig 1 and S2 Table). The average concentration of the top 30 metabolites in feces is shown in Table 5. The highest concentration of VFAs, such as acetate, propionate, and butyrate were in feces. The results were similar to those found in the ruminal fluid and many organic acid-related metabolites were identified. The metabolites in the fecal sample were involved in galactose metabolism; glyoxylate and dicarboxylate metabolism; amino sugar and nucleotide sugar metabolism; pentose phosphate pathway; glycolysis and gluconeogenesis; butanoate metabolism; neomycin, kanamycin, and gentamicin biosynthesis; and pentose and glucuronate interconversion (Fig 4E).

**Table 5:** Concentrations of the top 30 metabolites in feces by ^1^H-NMR analysis.

Metabolite	Concentration^1^	Metabolite	Concentration
*Alcohols*		*Lipids*	
Methanol	25.4 ± 4.9	Azelate	26.0 ± 7.6
Ethanol	19.95 ± 4.15	3-Methylglutarate	11.35 ± 3.05
*Amino acids and derivatives*		*Organic acids*	
Glutamate	20.7 ± 2.9	Acetate	2655.8 ± 953.8
*Carbohydrates*		Propionate	491.0 ± 205.9
Glucose	47.65 ± 10.12	Butyrate	189.3 ± 95.2
Ribose	44.65 ± 15.85	Valerate	65.26 ± 35.63
Galactose	29.76 ± 5.95	Isobutyrate	63.52 ± 13.81
Glucose-6-phosphate	24.2 ± 6.79	Isovalerate	36.4 ± 7.04
Glucitol	22.25 ± 6.05	Gluconate	20.3 ± 3.4
Xylose	21.86 ± 4.44	Formate	16.1 ± 1.71
Isocitrate	20.8 ± 2.59	*Others*	
Glucuronate	17.15 ± 7.35	Fucose	16.25 ± 6.55
Fructose	16.9 ± 5.5	Cellobiose	14.1 ± 3.5
Lactose	16.16 ± 2.73	4-Hydroxyphenyllactate	13.45 ± 3.55
Erythritol	15.25 ± 4.85	3-Phenylpropionate	11.83 ± 1.97
Lactulose	12.95 ± 5.05		
Galactonate	12.43 ± 2.06		

^1^ μM, Median ± interquartile range (n = 4).

### Common metabolites among five biofluids

The metabolite concentration across the biofluids is shown in Table 6 to visualize the differences between samples. In total, 23 metabolites were ubiquitous to all biofluids. Among high concentration common metabolites in the biofluids (rumen, serum, milk and urine), three, one, nine, and ten metabolites were identified, respectively. These metabolites included anserine, imidazole, and acetate in ruminal fluid; methanol in serum; O-phosphocholine, isocitrate, lactose, lactulose, N-acetylglucosamine, ribose, and 3-hydroxyisovalerate in milk; and histamine, 1-methylhistidine, 5-hydroxyindole-3-acetate, N-nitrosodimethylamine, succinylacetone, 3-methylhistidine, 4-pyridoxate, N-acetylserotonin, and pyridoxine in urine.

**Table 6:** Concentration of the metabolites common among the five biofluid identified by ^1^H-NMR.

Class	Metabolite	Concentration^1^					p-value^2^	-LOG(p)	FDR^3^
		Ruminal fluid	Serum	Milk	Urine	Feces			
*Alcohols*	Methanol	10.37 ± 0.87	96.17 ± 2.97	10.77 ± 0.74	22.05 ± 10.25	25.4 ± 4.9	***	8.50	***
*Aliphatic acylic compounds*	O-Phosphocholine	4.32 ± 1.48	3.05 ± 0.81	5.5 ± 0.1	1.96 ± 0.29	2.26 ± 0.27	***	2.38	***
*Amines*	Histamine	2.03 ± 0.29	1.2 ± 0.14	3.02 ± 0.39	18.52 ± 11.11	1.42 ± 0.37	***	7.81	***
*Amino acids*	1-Methylhistidine	2.37 ± 0.57	2.37 ± 0.37	4.02 ± 1.33	53.42 ± 44.98	1.85 ± 0.25	***	7.73	***
	Anserine	5.8 ± 1.76	2.15 ± 0.65	5.57 ± 1.72	4.72 ± 0.61	2.2 ± 0.47	***	6.00	***
*Carbohydrates*	Isocitrate	21.36 ± 3.06	15.05 ± 2.37	297.3 ± 40.4	116.3 ± 64.29	20.8 ± 2.59	**	1.78	**
	Lactose	38.75 ± 0.55	6.73 ± 1.44	43324.25 ± 3799.18	429.2 ± 148.96	16.16 ± 2.73	***	5.98	***
	Lactulose	12.55 ± 2.39	3.6 ± 0.1	103.55 ± 34.5	22.05 ± 10.25	12.95 ± 5.05	0.4885	0.31	0.4885
	N-Acetylglucosamine	15.4 ± 3.85	1.66 ± 0.62	126.95 ± 19.33	10.85 ± 6.85	10.57 ± 2.68	0.2872	0.54	0.3003
	Ribose	19.25 ± 0.65	17.66 ± 8.4	51.2 ± 5.1	31.9 ± 26.1	44.65 ± 15.85	**	1.70	**
*Carboxylic acids*	3-Hydroxyisovalerate	4.6 ± 1.31	3.57 ± 1.75	18.3 ± 6.11	6.03 ± 2.93	0.7 ± 0.1	*	1.12	*
*Imidazolinones*	Imidazole	9.72 ± 3.64	3.8 ± 0.4	7.4 ± 1.3	9.32 ± 3.21	3.05 ± 0.23	***	5.39	***
*Indoles*	5-Hydroxyindole-3-acetate	3.1 ± 0.79	0.96 ± 0.12	2.65 ± 0.05	24.96 ± 11.26	1.83 ± 0.58	***	2.83	***
*Lipids*	Choline	1.26 ± 0.66	0.63 ± 0.08	372.62 ± 39.23	1.65 ± 0.85	2.95 ± 1.01	***	3.60	***
*Organic acids*	Acetate	13664.22 ± 864.37	153.5 ± 13.06	31.85 ± 2.82	249.53 ± 80.82	2655.82 ± 953.78	***	10.16	***
	N-Nitrosodimethylamine	5.56 ± 0.03	0.9 ± 0.1	24.52 ± 1.36	36 ± 20.7	2.62 ± 0.7	**	1.66	**
	Succinylacetone	5.15 ± 1.12	2.6 ± 0.22	17 ± 2.72	26.7 ± 12.21	1.06 ± 0.23	***	4.52	***
*Others*	3-Methylhistidine	1.5 ± 0.11	3.65 ± 1.14	1.83 ± 0.31	36.22 ± 12.88	1.6 ± 0.2	***	8.41	***
	4-Pyridoxate	0.97 ± 0.08	1.4 ± 0.2	1.2 ± 0.1	7.4 ± 4.35	1.33 ± 0.58	***	4.86	***
	Betaine	1.26 ± 0.66	32.47 ± 1.92	190.42 ± 18.16	36.12 ± 17.66	2.85 ± 2.25	***	6.02	***
	Melatonin	5.1 ± 0.78	1.06 ± 0.2	3.26 ± 1.21	28.15 ± 26.95	3 ± 0.9	**	1.81	**
	N-Acetylserotonin	3.12 ± 0.54	0.7 ± 0.15	1.95 ± 0.35	15.6 ± 5.58	1.96 ± 0.43	***	3.36	***
	Pyridoxine	0.63 ± 0.18	0.55 ± 0.06	1.22 ± 0.04	2.93 ± 1.58	0.7 ± 0.1	***	3.39	***

^1^ μM, Median ± interquartile range (n = 4).

^2^ p-value: *** < 0.01, ** < 0.05, * < 0.1.

^3^ False discovery rate (FDR) controlled by the method of Benjanini-Hochberg: *** < 0.01, ** < 0.05, * < 0.1.

## Discussion

The analysis of metabolites in the ruminal fluid is important for understanding the ruminal microbial ecosystem. The rumen contains various microorganisms and ruminal fermentation owing to a variety of metabolites produced by these microorganisms. In particular, VFAs are the final product of anaerobic fermentation in the rumen and a major source of metabolizable energy in ruminants [22]. The three major VFAs in the ruminal fermentation system are acetate, propionate, and butyrate. The ratio of the various VFAs depends on the type of feedstuff fed to the ruminant. VFAs are absorbed through the ruminal wall and transported to the liver via the blood, where they are converted to other sources of energy for milk production, pregnancy, and growth [23]. VFAs, including 3-phenylpropionate, methylamine, valerate, isobutyrate, glucose, pimelate, fructose, and isovalerate had the highest concentration among the metabolites. Glucose and fructose were included as reference sugars [24]. 3-phenylpropionate has been identified as an aromatic acid in previous studies and ruminal microbes produce these metabolites from plant phenolic compounds by hydrogenation [25, 26], as this sugar is a minor component of the soluble fraction of plants. The rumen microbes can utilize all these sugars, including sucrose and fructose [27]. Methylamine is an amino acid produced in the process of rumen nitrogen metabolism. As the amount of grain feed increases, the amount produced increases. In addition, it is known as a biogenic amine such as tyramine and tryptamine, and is used for research on ruminal acidosis [26, 28]. The metabolite results obtained from the rumen could be useful for studying ruminal acidosis.

Blood plasma and serum contain a wide range of macromolecules that can overlap with peaks from small molecule metabolites in NMR under specific physiological and pathological states [29]. Large-scale metabolites in blood plasma and serum are a growing area of interest owing to their use in the diagnosis of human diseases. Metabolites of higher concentrations in serum can reflect changes in various organs in the body. Glucose, which is associated with glycosynthesis in metabolic processes in the muscle tissue [30], was also detected in the serum. Glucose is transformed into glycogen through glycosynthesis and stored as an energy source [31]. Acetate, related to the main precursors of milk fat synthesis, is transported from the rumen to the blood and mammary gland [32]. Creatine, creatinine, and alanine are metabolites involved in muscle metabolism [33]. Alanine is one of the most important amino acids in muscle synthesis. Creatine and creatinine are metabolites produced when muscle cells break down and are used in various studies as indicators of muscle mass [34]. 3-hydroxybutyrate and acetone are ketone-related metabolites that have been used in ketosis-related studies [35]. Ketosis is diagnosed by measuring the ketone bodies in blood and milk and occurs when ketone bodies from adipose tissue are used instead of glucose when the energy required by the body is insufficient. The analysis of metabolites in the blood may be useful in body weight-related muscle synthesis and ketosis studies related to malnutrition.

The composition of milk is influenced by a range of factors, such as diet, genetics, and number and stages of lactation, and additional factors, such as seasonal variation, somatic cell count, and milk processing [36–38]. Lactose is the most common carbohydrate in milk, accounting for 2–8% of the milk's weight. The accumulation of lactose is a major cause of osmotic pressure and the water attracted by the osmotic pressure makes up most of the water content of milk [39]. Guanidoacetate is an intermediate that converts glycine to creatine and methylated it to creatine in the blood [40]. It was relatively creatine deficiency syndromes and amino acid metabolism [41]. Glucitol, less commonly known as sorbitol, is an intermediate in the metabolism of fructose, mannose, and galactose. It has been used therapeutically as a laxative and in veterinary medicine for ruminant ketosis, as an osmotic diuretic [42]. Arabinitol (or arabitol) can be formed by the reduction of either arabinose or lyxose and indicates excessive growth of intestinal microbes, such as yeast and fungal species [43]. In a report by Thomas et al. a reduction in sn-glycero-3-phosphocholine, d-glycerol-1-phosphate, and glycerol phospholipid levels were observed in milk samples from cows with clinical and subclinical mastitis [44]. Choline is positively associated with good coagulation parameters, whereas carnitine, lactose, and citrate are negatively associated with choline [45]. The analysis of metabolites in urine can be employed in prospective studies of feed consumed by host animals and ruminant microbial protein production studies.

The monitoring of the concentration of certain metabolites in urine using metabolomics has become an important method for the early diagnosis of disease [37]. Owing to the less complex sample pre-treatment, lower protein content, and lower sample complexity, including fewer intermolecular interactions, urine as an analytical tool has a number of advantages over other biofluids. Urinary metabolomics approaches are used to screen for potential early diagnostic and prognostic biomarkers of disease. Hippurate, the glycine conjugate of benzoic acid, is a component of urine with a strong association to diet and the intestinal microbiota and typically increases with the increased consumption of phenolic compounds (e.g., tea, wine, and fruit juices) [46]. Urinary purine derivatives, such as allantoin and xanthine, have been widely used to estimate ruminal microbial protein production [47]. The principle is that the microbial marker duodenal purine base (PB) is effectively absorbed and the derivatives are mostly excreted by the kidneys. The proportion of PB in the urine closely reflects microbial protein flow and is predictable [48]. Allantoin is an oxidative end product of purines in mammals [49]. Xanthine is a purine base and an intermediate in the synthesis of guanosine monophosphate incorporated into RNA [50]. Lactose has been detected in the urine of lactating and non-lactating cows despite the low concentration [51]. Glycolate has been reported to be an important metabolite for the diagnosis of disease, as determined by NMR [52]. Trimethylamine N-oxide is a product of the oxidation of trimethylamine (derived from choline), a common metabolite in animals [53].

Many studies have focused on the metabolomic analysis of urine, plasma, and tissue biopsies, yet the analysis of fecal samples is at an early stage [54]. In the large intestine of the cow, microorganisms, including bacteria, protozoa, and fungi, have cellulase, protease, deaminase, and urease activities and release fermentation products, including VFAs and ammonia nitrogen [55]. Metabolites observed in feces were similar to those in the rumen, including organic acids and carbohydrates.

As in previous research, studies that compare treatments for a single or several biofluids are actively being conducted [7–11]. In addition, studies analyzing common metabolites in biological fluids using ^1^H-NMR have also been undertaken. A study on the metabolic profiles of yak (*Bos grunniens*) compared serum, feces, and urine, and revealed that 15 metabolites were ubiquitous across all biofluids [16]. Additionally, in the metabolic profile of a racehorse, a total of 102 metabolites were assigned across three biological matrices (plasma, urine, and feces), and a core metabolome of 14 metabolites was ubiquitous across all biofluids [17]. In the present study, we compared the metabolite concentrations of five biofluids and investigated the metabolites that are observed commonly. The most concentrated metabolites in the five biofluids were found to be related to some of the biological mechanisms. Some studies have also shown that metabolites with high concentration in ruminal fluid and feces are VFAs such as acetate, propionate, and butyrate, which are associated with microbial fermentation [56]. Increased amounts of carbohydrate in the large intestine during subacute ruminal acidosis can stimulate fermentation by bacteria and increase the VFAs of the feces [56]. The metabolites of citric acid play an important role as intermediates in cellular energy metabolism in the tricarboxylic acid cycle as well as in glycolysis and fatty acid synthesis, and are highly observed in milk among all the biofluids [57]. The urinary excretion of 1- and 3-methyl histidine in humans and livestock has been accepted as a measure of muscle protein breakdown and as a product of decomposition [58, 59]. Histamine is produced by microbial fermentation and is observed in various organs, tissues, and biofluids [60]. Among blood, milk, and urine, it is highly observed in urine [61]. The higher urinary histamine values of silage-fed animals undoubtedly reflect the higher initial histamine content of silage compared to that of roughage [62]. Melatonin is synthesized in the pineal gland and in many other organs in mammals, and the synthesis and secretion of melatonin are involved in the circadian rhythm [63, 64]. Several studies have reported that melatonin plays important roles in regulating adipose differentiation and fat synthesis [65, 66].

In this pilot study, although the number of animals used was small, simultaneous metabolic profiling of different biofluids was conducted to foster future research. Metabolites from fermented feedstuff in the ruminal fluid were identified and serum metabolites related to muscle metabolism were observed to be high. In milk, metabolites related to milk composition were identified and in urine, metabolites produced as a result of the metabolic processes in various organs were identified. Feces exhibited a complex array of fermentation products of microorganisms present in the large intestine, as well as those produced owing to body metabolism. Therefore, our study provides an understanding of the metabolite profiles of dairy cows, which can be developed for use in further research on dairy cows.

## Conclusions

Metabolites in the biological fluids of cows were screened using ^1^H-NMR and measured metabolites were ranked for each biofluids. The trends of metabolites in biofluids were distinguishable and the availability of metabolite was confirmed. Screening for metabolites in biofluids can be used in preliminary studies on various diseases and to investigate metabolic changes in biofluids and may be pertinent depending on the type of study. In addition, further research is needed as the metabolites may vary depending on various factors, such as the metabolic and physiological state of the animal, the composition of the feed, and individual differences.

## Supporting information

S1 Table The formulation and chemical composition of experiment diet. (DOCX)

S2 Table The detected of metabolite in biofluids. (DOCX)

S1 Fig Representative proton nuclear magnetic resonance spectral of five biofluids from dairy cows. (A) Ruminal fluid, (B) Serum, (C) Milk, (D) Urine, (E) Feces.
(TIF)

S2 Fig Permutation test statistics at 1000 permutations with observed statistic at p = 0.001. (TIF)
