# Supplementary material for: Exploration of metabolite profiles in the biofluids of dairy cows by proton nuclear magnetic resonance analysis
Source: PLoS One. 2021 Jan 29;16(1):e0246290. doi: 10.1371/journal.pone.0246290 (PMC7845951; doi:10.1371/journal.pone.0246290)
Supplement: S1 Table — (DOCX) [file pone.0246290.s001.docx]

**S1 Table.** The formulation and chemical composition of experiment diet

| Content | *% of DM* | Content | Concentrate | Italian ryegrass |
| --- | --- | --- | --- | --- |
| *Ingredients* |  | *Chemical composition (% of DM)* | | |
| Ground corn | 3.06 | Moisture | 11.20 | 7.40 |
| Ground wheat | 17.44 | Crude protein | 20.01 | 5.60 |
| Salt | 0.70 | Ether extract | 2.00 | 0.84 |
| Molasses | 3.50 | Crude ash | 6.43 | 4.84 |
| Soy hull | 5.36 | Crude fiber | 20.00 | 34.84 |
| Wheat flour | 20.10 | Ca | 0.80 | 0.21 |
| Rice bran | 0.84 | P | 0.51 | 0.13 |
| Corn DDG | 5.00 | Acid detergent fiber | 6.86 | 38.95 |
| DDGS | 15.00 | Neutral detergent fiber | 19.81 | 66.54 |
| Soybean meal | 6.12 |  |  |  |
| Urea | 0.51 |  |  |  |
| Sodium bicarbonate | 0.84 |  |  |  |
| Condensed molasses solubles | 1.50 |  |  |  |
| Corn gluten feed | 18.00 |  |  |  |
| Limestone | 1.27 |  |  |  |
| Palm oil | 0.39 |  |  |  |
| Flavor | 0.02 |  |  |  |
| Mineral/Vitamin premix^1^ | 0.35 |  |  |  |
| Total | 100.00 |  |  |  |

^1^Mineral & vitamin premix contained vit. A 2,650,000 IU, vit. D3 530,000 IU, vit. E 1,050 IU, niacin 10,000 mg, Mn 4,400 mg, Zn 4,400 mg, Fe 13,200 mg, Cu 2,200 mg, iodine 440 mg, and Co, 440 mg/kg of Grobic-DC provided from Bayer Health Care (Leverkusen, Germany).

DDG: Distillers dried grains, DDGS: Distillers dried grains with solubles.
