# Supplementary material for: Exploration of metabolite profiles in the biofluids of dairy cows by proton nuclear magnetic resonance analysis
Source: PLoS One. 2021 Jan 29;16(1):e0246290. doi: 10.1371/journal.pone.0246290 (PMC7845951; doi:10.1371/journal.pone.0246290)
Supplement: S2 Table — (DOCX) [file pone.0246290.s002.docx]

**S2 Table. The detected of metabolite in biofluids**

| **Class** | **Metabolite** | **Sample^1)^** | **Formula** | **Class** | **Metabolite** | **Sample** | **Formula** |
| --- | --- | --- | --- | --- | --- | --- | --- |
| *Carbohydrates* | 1,3-Dihydroxyacetone | R, F | C_3_H_6_O_3_ | *Carboxylic acids* | Guanidoacetate | S, M, U, F | C_3_H_7_N_3_O_2_ |
| *Others* | 1,3-Dimethylurate | U | C_7_H_8_N_4_O_3_ | *Amino acids and derivatives* | Hippurate | U | C_9_H_9_NO_3_ |
| *Others* | 1,6-Anhydro-beta-D-glucose | U | C_6_H_10_O_5_ | *Amines* | Histamine | ALL | C_5_H_9_N_3_ |
| *Others* | 1,7-Dimethylxanthine | R, S, U, F | C_7_H_8_N_4_O_2_ | *Amino acids* | Histidine | U | C_6_H_9_N_3_O_2_ |
| *Amino acids* | 1-Methylhistidine | ALL | C_7_H_11_N_3_O_2_ | *Benzoic acids* | Homogentisate | F | C_8_H_8_O_4_ |
| *Nucleosides, Nucleotides* | 2'-Deoxyuridine | R | C_9_H_12_N_2_O_5_ | *Carboxylic acids* | Homovanillate | R, M, U, F | C_9_H_10_O_4_ |
| *Amino acids* | 2-Furoylglycine | M | C_7_H_7_NO_4_ | *Carboxylic acids* | Hydroxyacetone | S, M, F | C_3_H_6_O_2_ |
| *Lipids* | 2-Hydroxy-3-methylvalerate | R, F | C_6_H_12_O_3_ | *Others* | Ibuprofen | R, M, U, F | C_13_H_18_O_2_ |
| *Carboxylic acids* | 2-Hydroxyisobutyrate | S, F | C_4_H_8_O_3_ | *Imidazolinones* | Imidazole | ALL | C_3_H_4_N_2_ |
| *Lipids* | 2-Hydroxyisocaproate | U | C_6_H_12_O_3_ | *Others* | Indole-3-acetate | R, U | C_10_H_9_NO_2_ |
| *Others* | 2-Hydroxyphenylacetate | R, U, F | C_8_H_8_O_3_ | *Others* | Indole-3-lactate | U | C_11_H_11_NO_3_ |
| *Lipids* | 2-Hydroxyvalerate | S, U | C_5_H_10_O_3_ | *Nucleosides, Nucleotides* | Inosine | M | C_10_H_12_N_4_O_5_ |
| *Others* | 2-Oxocaproate | M | C_6_H_10_O_3_ | *Organic acids* | Isobutyrate | R, F | C_4_H_8_O_2_ |
| *Organic acids* | 2-Oxoglutarate | M | C_5_H_6_O_5_ | *Carbohydrates* | Isocitrate | ALL | C_6_H_8_O_7_ |
| *Organic acids* | 2-Oxoisocaproate | F | C_6_H_10_O_3_ | *Benzoic acids* | Isoeugenol | M, F | C_10_H_12_O_2_ |
| *Benzoic acids* | 3,4-Dihydroxybenzeneacetate | F | C_8_H_8_O_4_ | *Amino acids and derivatives* | Isoleucine | R, S, U, F | C_6_H_13_NO_2_ |
| *Benzoic acids* | 3,4-Dihydroxymandelate | U | C_8_H_8_O_5_ | *Alcohols* | Isopropanol | R, F | C_3_H_8_O |
| *Lipids* | 3,5-Dibromotyrosine | R, U | C_9_H_9_Br_2_NO_3_ | *Organic acids* | Isovalerate | R, F | C_5_H_10_O_2_ |
| *Carboxylic acids* | 3-Chlorotyrosine | U | C_9_H_10_ClNO_3_ | *Others* | Kynurenate | U | C_10_H_7_NO_3_ |
| *Lipids* | 3-Hydroxy-3-methylglutarate | R, S, U, F | C_6_H_10_O_5_ | *Amines* | Kynurenine | R, M | C_10_H_12_N_2_O_3_ |
| *Lipids* | 3-Hydroxybutyrate | R, S | C_4_H_8_O_3_ | *Organic acids* | Lactate | S | C_3_H_6_O_3_ |
| *Carboxylic acids* | 3-Hydroxyisovalerate | ALL | C_5_H_10_O_3_ | *Carbohydrates* | Lactose | ALL | C_12_H_22_O_11_ |
| *Organic acids* | 3-Hydroxykynurenine | R | C_10_H_12_N_2_O_4_ | *Carbohydrates* | Lactulose | ALL | C_12_H_22_O_11_ |
| *Benzoic acids* | 3-Hydroxymandelate | U, F | C_8_H_8_O_4_ | *Amino acids and derivatives* | Leucine | S, F | C_6_H_13_NO_2_ |
| *Carboxylic acids* | 3-Hydroxyphenylacetate | R, M, U, F | C_8_H_8_O_3_ | *Others* | Levulinate | S, F | C_5_H_8_O_3_ |
| *Indoles* | 3-Indoxylsulfate | U | C_8_H_7_NO_4_S | *Organic acids* | Malate | R, U | C_4_H_6_O_5_ |
| *Lipids* | 3-Methylglutarate | R, U, F | C_6_H_10_O_4_ | *Carboxylic acids* | Malonate | S, M, U, F | C_3_H_4_O_4_ |
| *Others* | 3-Methylhistidine | ALL | C_7_H_11_N_3_O_2_ | *Carbohydrates* | Maltose | R, M, U | C_12_H_22_O_11_ |
| *Others* | 3-Methylxanthine | R, M, U, F | C_6_H_6_N_4_O_2_ | *Benzoic acids* | Mandelate | R, S, U, F | C_8_H_8_O_3_ |
| *Others* | 3-Phenylpropionate | R, U, F | C_9_H_10_O_2_ | *Carbohydrates* | Mannose | S, M, F | C_6_H_12_O_6_ |
| *Benzoic acids* | 4-Hydroxy-3-methoxymandelate | S, M, U, F | C_9_H_10_O_5_ | *Others* | Melatonin | ALL | C_13_H_16_N_2_O_2_ |
| *Benzoic acids* | 4-Hydroxyphenylacetate | R, U, F | C_8_H_8_O_3_ | *Alcohols* | Methanol | ALL | CH_4_O |
| *Others* | 4-Hydroxyphenyllactate | F | C_9_H_10_O_4_ | *Amino acids and derivatives* | Methionine | R, S, M, F | C_5_H_11_NO_2_S |
| *Others* | 4-Pyridoxate | ALL | C_8_H_9_NO_4_ | *Amines* | Methylamine | R, U, F | CH_5_N |
| *Pyridines* | 5,6-Dihydrothymine | S | C_5_H_8_N_2_O_2_ | *Others* | Methylguanidine | F | C_2_H_7_N_3_ |
| *Carboxylic acids* | 5-Aminolevulinate | S, M, U, F | C_5_H_9_NO_3_ | *Lipids* | Methylsuccinate | S, U, F | C_5_H_8_O_4_ |
| *Indoles* | 5-Hydroxyindole-3-acetate | ALL | C_10_H_9_NO_3_ | *Carboxylic acids* | N,N-Dimethylformamide | U, F | C_3_H_7_NO |
| *Indoles* | 5-Hydroxytryptophan | F | C_11_H_12_N_2_O_3_ | *Carboxylic acids* | N,N-Dimethylglycine | U, F | C_4_H_9_NO_2_ |
| *Benzoic acids* | 5-Methoxysalicylate | F | C_8_H_8_O_4_ | *Carboxylic acids* | N6-Acetyllysine | M, U, F | C_8_H_16_N_2_O_3_ |
| *Benzoic acids* | Acetaminophen | R, S, M, F | C_8_H_9_NO_2_ | *Carboxylic acids* | N-Acetylaspartate | F | C_6_H_9_NO_5_ |
| *Organic acids* | Acetate | ALL | C_8_H_8_O_3_ | *Carboxylic acids* | N-Acetylcysteine | R, F | C_5_H_9_NO_3_S |
| *Carbohydrates* | Acetoacetate | R, S, U, F | C_4_H_6_O_3_ | *Carbohydrates* | N-Acetylglucosamine | ALL | C_8_H_15_NO_6_ |
| *Others* | Acetoin | U | C_4_H_8_O_2_ | *Amino acids and derivatives* | N-Acetylglutamate | R, M, F | C_7_H_11_NO_5_ |
| *Others* | Acetone | R, S, U, F | C_3_H_6_O_3_ | *Carboxylic acids* | N-Acetylglycine | R, F | C_4_H_7_NO_3_ |
| *Benzoic acids* | Acetylsalicylate | M, U | C_9_H_8_O_4_ | *Carboxylic acids* | N-Acetylornithine | R | C_7_H_14_N_2_O_3_ |
| *Amino acids and derivatives* | Alanine | R, S, M, U | C_3_H_7_NO_2_ | *Others* | N-Acetylserotonin | ALL | C_12_H_14_N_2_O_2_ |
| *Imidazolinones* | Allantoin | M, U | C_4_H_6_N_4_O_3_ | *Carboxylic acids* | N-Acetyltyrosine | M, U | C_11_H_13_NO_4_ |
| *Carboxylic acids* | Alloisoleucine | S | C_6_H_13_NO_2_ | *Carboxylic acids* | N-alpha-Acetyllysine | R | C_8_H_16_N_2_O_3_ |
| *Amino acids* | Anserine | ALL | C_10_H_16_N_4_O_3_ | *Carboxylic acids* | N-Carbamoylaspartate | M | C_5_H_8_N_2_O_5_ |
| *Carbohydrates* | Arabinitol | M | C_5_H_12_O_5_ | *Others* | N-Methylhydantoin | U | C_4_H_6_N_2_O_2_ |
| *Others* | Arabinose | S, U, F | C_5_H_10_O_5_ | *Organic acids* | N-Nitrosodimethylamine | ALL | C_2_H_6_N_2_O |
| *Others* | Ascorbate | S, U | C_6_H_8_O_6_ | *Amino acids* | N-Phenylacetylglycine | R, S, U, F | C_10_H_11_NO_3_ |
| *Nucleosides, Nucleotides* | ATP | M | C_10_H_16_N_5_O_13_P_3_ | *Carboxylic acids* | N-Phenylacetylphenylalanine | R | C_17_H_17_NO_3_ |
| *Lipids* | Azelate | F | C_9_H_16_O_4_ | *Lipids* | O-Acetylcarnitine | R, M, F | C_9_H_18_NO_4_ |
| *Others* | Betaine | ALL | C_5_H_11_NO_2_ | *Organic acids* | O-Acetylcholine | R, M, U, F | C_7_H_16_NO_2_ |
| *Others* | Biotin | R, M, U | C_10_H_16_N_2_O_3_S | *Benzoic acids* | o-Cresol | R, M, U, F | C_7_H_8_O |
| *Others* | Butanone | F | C_4_H_8_O | *Aliphatic acylic compounds* | O-Phosphocholine | ALL | C_5_H_15_NO_4_P |
| *Organic acids* | Butyrate | R, M, F | C_4_H_8_O_2_ | *Amino acids and derivatives* | Ornithine | S | C_5_H_12_N_2_O_2_ |
| *Others* | Caffeine | R, M, U, F | C_8_H_10_N_4_O_2_ | *Others* | Oxypurinol | R, U | C_5_H_4_N_4_O_2_ |
| *Lipids* | Caprate | R | C_10_H_20_O_2_ | *Carboxylic acids* | Pantothenate | R, S, U, F | C_9_H_17_NO_5_ |
| *Lipids* | Carnitine | R, S, M | C_7_H_15_NO_3_ | *Benzoic acids* | p-Cresol | R, U, F | C_7_H_8_O |
| *Amines* | Carnosine | U, F | C_9_H_14_N_4_O_3_ | *Organic acids* | Phenylacetate | R, U, F | C_8_H_8_O_2_ |
| *Others* | Cellobiose | S, M, U, F | C_12_H_22_O_11_ | *Lipids* | Pimelate | R | C_7_H_12_O_4_ |
| *Lipids* | Cholate | U | C_24_H_40_O_5_ | *Organic acids* | Propionate | R, F | C_3_H_6_O_2_ |
| *Lipids* | Choline | ALL | C_5_H_14_NO | *Others* | Pyridoxine | ALL | C_8_H_11_NO_3_ |
| *Carboxylic acids* | cis-Aconitate | M, U | C_6_H_6_O_6_ | *Carbohydrates* | Pyruvate | R, S, F | C_3_H_4_O_3_ |
| *Lipids* | Citraconate | U | C_5_H_6_O_4_ | *Others* | Riboflavin | R, M, U | C_17_H_20_N_4_O_6_ |
| *Carbohydrates* | Citrate | S, U, F | C_6_H_8_O_7_ | *Carbohydrates* | Ribose | ALL | C_5_H_10_O_5_ |
| *Amino acids* | Creatine | S, M, U, F | C_4_H_9_N_3_O_2_ | *Organic acids* | Salicylate | U | C_7_H_6_O_3_ |
| *Carboxylic acids* | Creatine phosphate | S, M, U, F | C_4_H_10_N_3_O_5_P | *Benzoic acids* | Salicylurate | R, U | C_9_H_9_NO_4_ |
| *Imidazolinones* | Creatinine | S, M, U, F | C_4_H_7_N_3_O | *Amines* | Sarcosine | S, M, F | C_3_H_7_NO_2_ |
| *Others* | Desaminotyrosine | U | C_9_H_10_O_3_ | *Lipids* | Sebacate | M, U | C_10_H_18_O_4_ |
| *Others* | Dimethyl sulfone | R, M, U, F | C_2_H_6_O_2_S | *Amines* | Serotonin | R, U | C_10_H_12_N_2_O |
| *Amines* | Dimethylamine | R, U, F | C_2_H_7_N | *Others* | sn-Glycero-3-phosphocholine | S, M, U, F | C_8_H_21_NO_6_P |
| *Carbohydrates* | Erythritol | R, F | C_4_H_10_O_4_ | *Carbohydrates* | Succinate | R, U, F | C_4_H_6_O_4_ |
| *Alcohols* | Ethanol | F | C_2_H_6_O | *Organic acids* | Succinylacetone | ALL | C_7_H_10_O_4_ |
| *Lipids* | Ethylene glycol | F | C_2_H_6_O_2_ | *Carbohydrates* | Sucrose | S | C_12_H_22_O_11_ |
| *Organic acids* | Ferulate | R, M, U, F | C_10_H_10_O_4_ | *Benzoic acids* | Syringate | R, S, U, F | C_9_H_10_O_5_ |
| *Organic acids* | Formate | S, U, F | CH_2_O_2_ | *Benzoic acids* | Tartrate | M, F | C_4_H_6_O_6_ |
| *Carbohydrates* | Fructose | R, U, F | C_6_H_12_O_6_ | *Others* | Theophylline | R, M, U, F | C_7_H_8_N_4_O_2_ |
| *Others* | Fucose | S, M, U, F | C_6_H_12_O_5_ | *Carbohydrates* | Threonate | R | C_4_H_8_O_5_ |
| *Organic acids* | Fumarate | M, U | C_4_H_4_O_4_ | *Amino acids* | Threonine | F | C_4_H_9_NO_3_ |
| *Others* | Galactarate | S, U, F | C_6_H_10_O_8_ | *Nucleosides, Nucleotides* | Thymidine | U | C_10_H_14_N_2_O_5_ |
| *Carbohydrates* | Galactitol | F | C_6_H_14_O_6_ | *Lipids* | Thymol | R, S, U, F | C_10_H_14_O |
| *Carbohydrates* | Galactonate | F | C_6_H_12_O_7_ | *Carboxylic acids* | trans-Aconitate | R, M, U, F | C_6_H_6_O_6_ |
| *Carbohydrates* | Galactose | M, F | C_6_H_12_O_6_ | *Carbohydrates* | Trehalose | M, F | C_12_H_22_O_11_ |
| *Benzoic acids* | Gallate | U | C_7_H_6_O_5_ | *Amines* | Trimethylamine | R, M, U | C_3_H_9_N |
| *Benzoic acids* | Gentisate | R, U | C_7_H_6_O_4_ | *Aliphatic acylic compounds* | Trimethylamine N-oxide | S, M, U, F | C_3_H_9_NO |
| *Carbohydrates* | Glucarate | M | C_6_H_10_O_8_ | *Amino acids and derivatives* | Tryptophan | U | C_11_H_12_N_2_O_2_ |
| *Carbohydrates* | Glucitol | M, F | C_6_H_14_O_6_ | *Amino acids and derivatives* | Tyrosine | U | C_9_H_11_NO_3_ |
| *Organic acids* | Gluconate | R, S, M, F | C_6_H_12_O_7_ | *Nucleosides, Nucleotides* | UDP-N-Acetylglucosamine | R, M | C_17_H_27_N_3_O_17_P_2_ |
| *Carbohydrates* | Glucose | R, S, F | C_6_H_12_O_6_ | *Nucleosides, Nucleotides* | Uracil | R | C_4_H_4_N_2_O_2_ |
| *Carbohydrates* | Glucose-6-phosphate | R, U, F | C_6_H_13_O_9_P | *Aliphatic acylic compounds* | Urea | M, U | CH_4_N_2_O |
| *Carbohydrates* | Glucuronate | M, U, F | C_6_H_10_O_7_ | *Nucleosides, Nucleotides* | Uridine | F | C_9_H_12_N_2_O_6_ |
| *Amino acids and derivatives* | Glutamate | F | C_5_H_9_NO_4_ | *Organic acids* | Valerate | R, F | C_5_H_10_O_2_ |
| *Amino acids* | Glutamine | S | C_5_H_10_N_2_O_3_ | *Amino acids and derivatives* | Valine | S, M, F | C_5_H_11_NO_2_ |
| *Lipids* | Glutaric acid monomethyl ester | F | C_6_H_10_O_4_ | *Benzoic acids* | Vanillate | R, M, U | C_8_H_8_O_4_ |
| *Carbohydrates* | Glutathione | F | C_10_H_17_N_3_O_6_S | *Nucleosides, Nucleotides* | Xanthine | U, F | C_5_H_4_N_4_O_2_ |
| *Amino acids and derivatives* | Glycine | S, M, F | C_2_H_5_NO_2_ | *Amino acids and derivatives* | Xanthurenate | R, U, F | C_10_H_7_NO_4_ |
| *Lipids* | Glycolate | S, U, F | C_2_H_4_O_3_ | *Carbohydrates* | Xylitol | S, F | C_5_H_12_O_5_ |
| *Carboxylic acids* | Glycylproline | R, S, U, F | C_7_H_12_N_2_O_3_ | *Carbohydrates* | Xylose | M, F | C_5_H_10_O_5_ |

^1)^ R, Rumen fluid; S, Serum; M, Milk; U, Urine; F, Feces.
