# Supplementary figures and images for: Exploration of metabolite profiles in the biofluids of dairy cows by proton nuclear magnetic resonance analysis
Source: PLoS One. 2021 Jan 29;16(1):e0246290. doi: 10.1371/journal.pone.0246290 (PMC7845951; doi:10.1371/journal.pone.0246290)

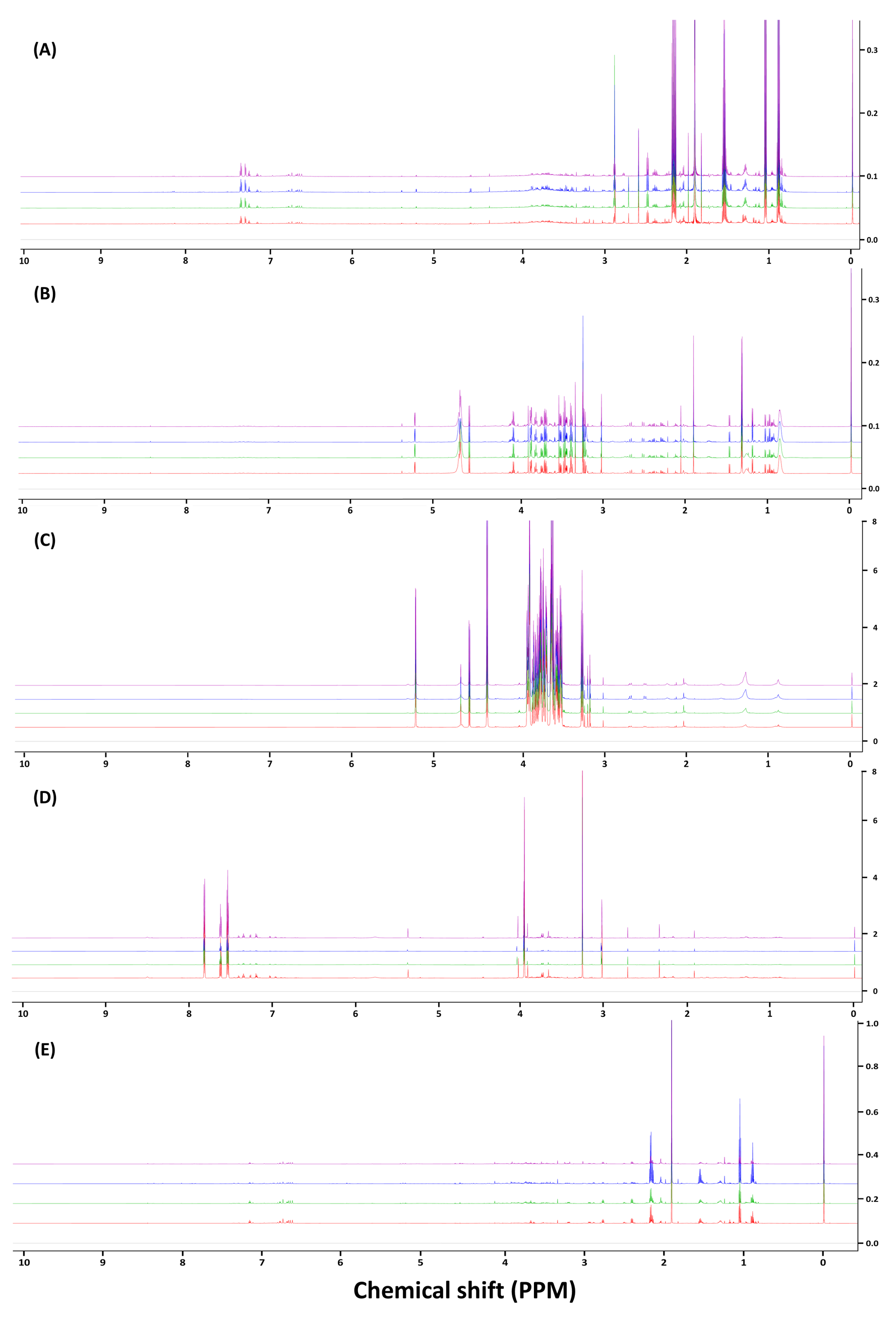

Supplement: S1 Fig — (A) Ruminal fluid, (B) Serum, (C) Milk, (D) Urine, (E) Feces. (TIF) [file pone.0246290.s003.tif]

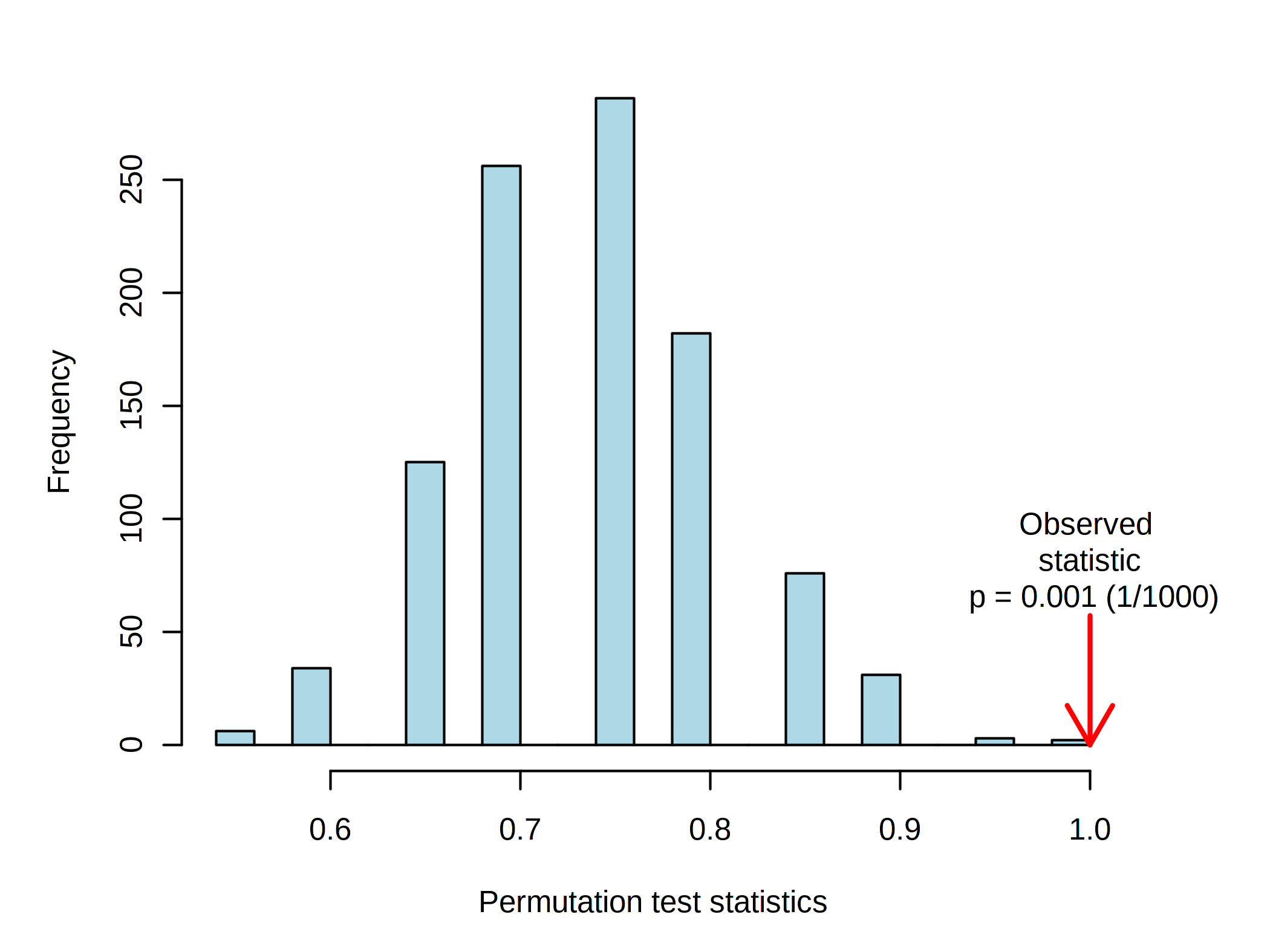

Supplement: S2 Fig — (TIF) [file pone.0246290.s004.tif]
